# From environment to brain: the role of microplastics in neurobehavioral disorders

**DOI:** 10.3389/fnins.2025.1691461

**Published:** 2025-12-01

**Authors:** Shan Zhang, Jianbing Wu, Mei Wu, Yuan Qin, Nan Zhu, Yijia Shen, Rong Chen, Zhujun Chen

**Affiliations:** Zhangjiagang Center for Disease Control and Prevention, Zhangjiagang, China

**Keywords:** microplastics, nanoplastics, neurobehavioral disorders, neurotoxicity, environmental effects

## Abstract

In recent years, the pervasive presence of microplastics has attracted significant attention from the scientific community, particularly concerning their potential implications for human health. Current literature suggests that microplastics may adversely affect the nervous system, with emerging evidence linking them to neurobehavioral disorders. However, many questions remain regarding the pathways of their environmental exposure, the specific effects on neurobehavior, and the underlying mechanisms of their impact. This review aims to explore the routes through which humans are exposed to microplastics, monitor behavioral changes associated with microplastic exposure, and examine how these particles infiltrate the body and traverse the blood-brain barrier. Several perspectives will be considered in assessing the potential mechanisms by which microplastics may influence neurobehavioral disorders, including oxidative stress, neurotransmitter regulation, and neuroplasticity. The article concludes by summarizing the effects of microplastics on neurobehavioral disorders, such as neurodegeneration and mood disorders, while analyzing the latest research findings. The primary objective of this study is to elucidate the neurotoxic effects of microplastics and their potential biological mechanisms, as well as to provide new insights and recommendations for future research in this domain.

## 1 Introduction

Microplastics (MPs) and nanoplastics (NPs) represent a class of environmental contaminants of increasing concern due to their pervasive presence and potential to impact human health. Defined as plastic particles measuring less than 5 μm and 100 nm in diameter, respectively, they are distinct from macroplastics (>5 mm), which are visible debris often excluded from MPs/NPs toxicology (Baroni et al., 2025). These small particles mainly originate from the breakdown of larger plastic items or industrial manufacturing releases, with common sources including personal care products, synthetic fibers, and packaging materials (Gamage and Mahagamage, 2024). Notably, MPs can accumulate in tissues such as the brain even at submicron sizes (Sun M. et al., 2024). NPs generally exhibit higher bioavailability and toxicity, entering the body via ingestion, inhalation, or dermal contact (Fraissinet et al., 2024), although size classifications vary—some studies define NPs as 1–100 nm, while others use <1 μm for practicality (Baroni et al., 2025; Cobanoglu et al., 2021). There is no universal harmful size threshold; effects depend on the biological model, dose, and particle properties, with reported thresholds ranging from tens of nanometers to several hundred nanometers (Araujo et al., 2025; Sun and Song, 2025). Research indicates that MPs have been extensively identified across various ecosystems, including marine, freshwater, and terrestrial environments, with concentrations in certain regions reaching concerning levels. Infiltration of MPs has been found in even the most secluded ecosystems, such as a remote high-altitude lake in the Tibetan Plateau in China, with MPs reaching as high as 5.56 items L^–1^ in surface water (Liang T. et al., 2024). This widespread environmental contamination poses a dual threat, endangering aquatic organisms and potentially impacting human health through the food chain, particularly seafood consumption (Pan Z. et al., 2022).

The pervasive environmental presence of MPs and NPs has escalated concerns for human health, with the brain being a potentially vulnerable target. These particles are now recognized as a novel environmental stressor of the nervous system. Key studies demonstrate their ability to penetrate the blood-brain barrier (BBB) (Adamiak et al., 2025) and, upon entry, to instigate fundamental neuropathological processes, including oxidative stress (Marcellus et al., 2024), Impaired Cellular Housekeeping (Cobanoglu et al., 2021), and synaptic dysfunction (Jin et al., 2022). This has led to the hypothesis that MPs exposure could be a potential environmental risk factor for disorders such as anxiety, cognitive deficits, and neurodevelopmental conditions, though establishing direct causal links in humans requires further investigation.

Due to their minuscule size, MPs and NPs are hypothesized to traverse biological barriers, including the blood-brain barrier, infiltrate cellular interiors, and potentially exert direct cytotoxic effects (Wu et al., 2023). For instance, an *in vitro* study using human neural stem cell line reported oxidative stress, DNA damage and apoptosis after 4 days of exposure to 30 nm polystyrene (PS) nanoparticles, observations which may correlate with potential pathway for cellular damage and neurodevelopmental disorders (Martin-Folgar et al., 2024). Furthermore, MPs can act as vectors for other neurotoxicants, thereby potentiating their harm. *In vivo* studies using marine medaka and zebrafish have shown they enhance the bioavailability and neurotoxicity of pollutants like tributyltin and polychlorinated biphenyls, leading to increased neurodevelopmental anomalies and impaired locomotor behavior (Lin et al., 2024; Varshney et al., 2024). Supporting these mechanistic insights, preliminary animal studies indicate that maternal exposure to MPs during gestation and lactation might adversely affect the neurodevelopment of offspring, resulting in behavioral deficits such as increased anxiety and impaired spatial memory (Tian et al., 2024). It is critical to acknowledge, however, that these findings primarily originate from model systems, and extrapolating them directly to human health outcomes remains a key challenge.

Neurobehavioral disorders, including anxiety, depression, cognitive impairment, autism spectrum disorder (ASD), and attention deficit hyperactivity disorder (ADHD), represent a significant global health burden. Anxiety, depression and cognitive impairment primarily affect emotional wellbeing and cognitive function, often stemming from neurobiological dysregulations such as neurotransmitter imbalances and neuroendocrine disruptions. Mood disorders are highly prevalent, affecting approximately one in seven individuals lifetime (Brown et al., 2018) and fine particulate matter exposure in the sleep environment has been linked to an increased risk of adult cognitive impairment (Pan R. et al., 2022). ASD is characterized by impairments in social interaction and communication, alongside repetitive behavioral patterns (Candini, 2024). ADHD, marked by inattention, hyperactivity, and impulsivity, has a prevalence of 3.4% in adults and up to 7% in children, significantly hindering learning and daily life (Fayyad et al., 2007). Their etiology is complex, involving an interplay of genetic predisposition and environmental factors. Recent scholarly investigations have increasingly implicated environmental contaminants in the onset and progression of neurobehavioral disorders. In this context, MPs have emerged as a novel environmental stressor of concern. It is hypothesized that MPs may alter the brain’s neurophysiological environment, thereby potentially influencing the risk or severity of neurobehavioral disorders (Liu et al., 2024). Establishing and validating this potential link constitutes a critical frontier in environmental health science.

In light of the increasing global prevalence of MPs pollution, this review explores the association between MPs and neurobehavioral disorders. Through a systematic analysis of existing literature, we aim to clarify the mechanistic pathways through which MPs interact with neurological processes and inform the development of targeted public health strategies and environmental regulations.

## 2 Assessment of human exposure to MPs

Human exposure to microplastics occurs through multiple pathways, primarily ingestion, inhalation, and dermal contact. MPs infiltrate the food chain via filter-feeding marine organisms, such as mussels and oysters, subsequently accumulating in consumable seafood products, including fish and shellfish (Masia et al., 2022). In European nations with significant crustacean and bivalve consumption, such as Belgium and the Netherlands, individuals may ingest as many as 11,000 microplastic particles annually through seafood consumption (Van Cauwenberghe et al., 2015). Additionally, drinking water serves as a critical source of MPs exposure. The phenomenon of modern seawater intrusion facilitates the migration of MPs from seawater to coastal groundwater, potentially leading to severe microplastic contamination in these aquifers (Chen G. et al., 2024). Common terrestrial food items, including salt, honey, beer, and bottled water, have also been found to contain MPs. For instance, the concentration of MPs in sea salt has been reported to range from 550 to 681 particles per kilogram, while bottled water contains an average of 325 microplastic particles per liter (Mason et al., 2018). Furthermore, polypropylene (PP) and polyethylene (PE) MPs have been identified in human feces, indicating their entry into the human body via the digestive system (Schwabl et al., 2019; Zhang et al., 2021). A study conducted by Peking University detected PS, PE, and polyvinyl chloride (PVC) MPs in human prostate tumor tissues, with their prevalence correlating with the frequency of take-out food consumption among patients (Deng et al., 2024). This finding suggests a potential link between the long-term accumulation of plastic materials in the body and their degradation.

Microplastic particles present in the air can also enter the human body through inhalation. These airborne MPs primarily originate from waste incineration, tire wear, and the release of particles from building materials (Dris et al., 2016). Urban atmospheres in China exhibit relatively high concentrations of MPs, largely attributable to the degradation of plastic products and industrial emissions. In Shanghai, for example, air concentrations of MPs range from 0 to 4.18 particles per cubic meter, with each resident inhaling approximately 21 microplastic particles daily from outdoor environments. It is estimated that around 120.7 kg of MPs are dispersed into the air in Shanghai annually (Liu et al., 2019). The concentration of MPs is closely associated with urban industrial activities, and particles with diameters less than 20 μm can penetrate deeply into the lungs, potentially eliciting inflammatory responses. Workers in plastic manufacturing environments face a 3.6-fold increase in the risk of respiratory symptoms (Atis et al., 2005). Individuals engaging in light physical activity may inhale approximately 272 microplastic particles daily, with these minuscule particles capable of penetrating the alveoli and entering the bloodstream (Leslie et al., 2022).

**FIGURE 1:**
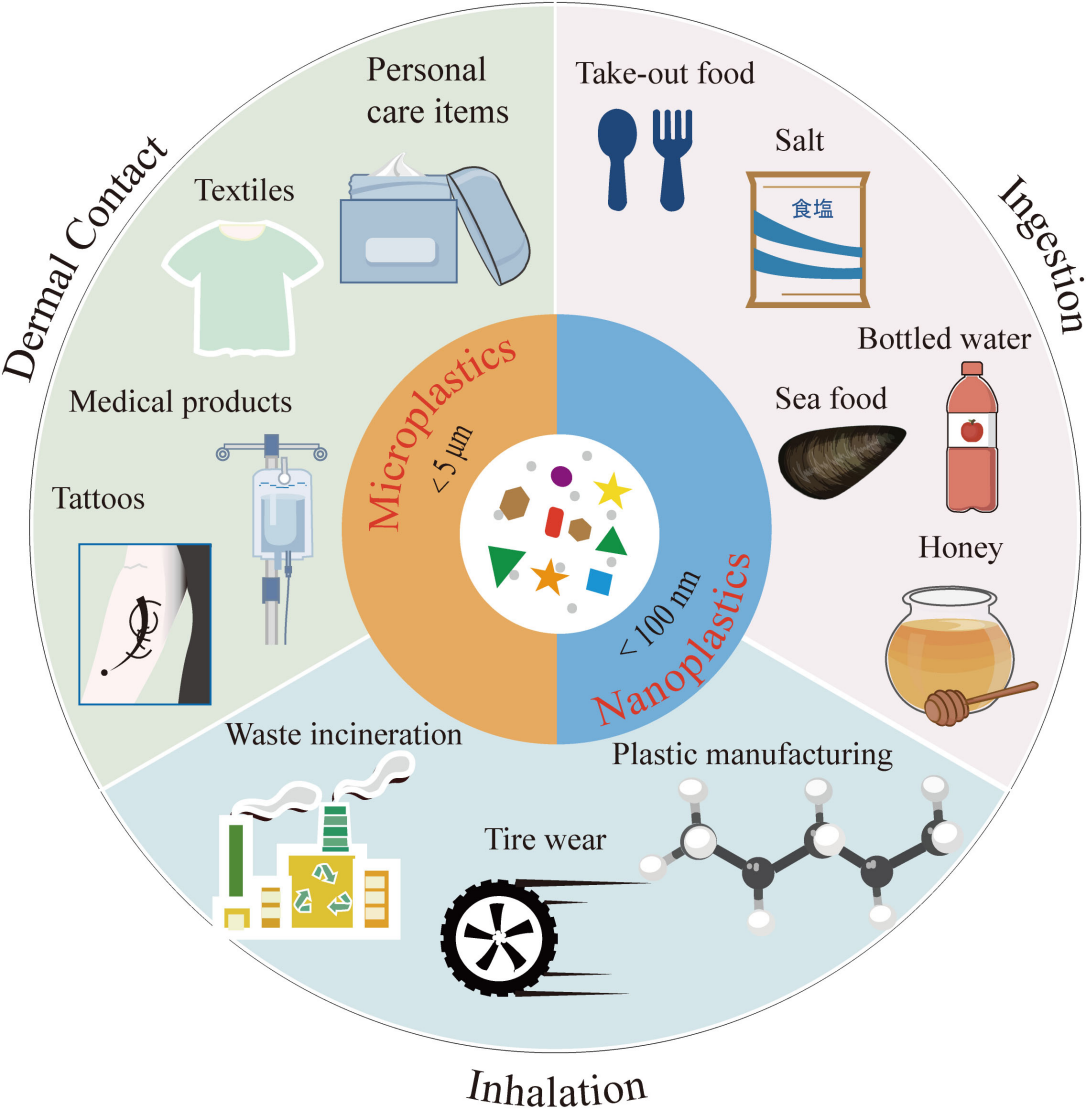
The exposure pathways of microplastics within the human body.

The low surface area-to-body ratio of microplastic particles facilitates their absorption through direct skin contact, particularly when using cosmetics that contain MPs. Plastic microbeads found in products such as toothpaste and facial scrubs can be absorbed through skin fissures or sweat glands, while NPs may penetrate the dermis (Leslie et al., 2022). Synthetic fiber garments release a significant quantity of microplastic fibers during laundering, with each wash cycle yielding over 1,900 fibers; notably, woolen garments release 180% more fibers than other clothing types (Browne et al., 2011). These fibers may enter the body through dermal contact or inhalation, and prolonged use of synthetic fiber clothing may result in continuous exposure. Research indicates that tattoo procedures can directly introduce numerous microplastic particles into the skin, where they may persist for extended periods and potentially migrate to lymph nodes and other organs (Schreiver et al., 2017). Additionally, medical plastic products, such as catheters and packaging materials, represent another pathway for exposure. A study from Fudan University (Huang et al., 2025) revealed that even after filtration, intravenous infusion bottles made from PP contain approximately 7,500 microplastic particles per liter (with particle sizes ranging from 1 to 62 μm). These particles can directly enter the human circulatory system through intravenous infusion, depositing in organs such as the lungs, liver, and spleen, and may provoke inflammatory responses. The exposure pathways of MPs within the human body are illustrated in Figure 1.

Evidence of human internal exposure to MPs is mounting from biomonitoring studies. A multi-city study involving university students in China found MPs in 98.7% of fecal samples, with PE, PVC, and polycarbonate (PC) being the most prevalent polymers detected. Statistical analyses revealed significant correlations between fecal microplastic loads and two behavioral factors: the consumption of bottled water and the habitual use of heated plastic food containers (Song et al., 2025). The internal exposure level of MPs in serum (20.81 μg/g) was found to be higher than that in urine (5.06 μg/g), suggesting that serum MPs levels may serve as a more reliable indicator for assessing long-term exposure (Song et al., 2024). Of particular concern are findings from maternal-fetal studies, which have detected MPs in both amniotic fluid and placental tissue, with particle counts being significantly higher in placental samples (Halfar et al., 2023). This differential distribution suggests that the placenta may act as a partial barrier against fetal exposure, although it is not entirely impermeable. Analysis of exposure pathways indicates that maternal MPs intake primarily arises from the use of plastic bottles and abrasive personal care products (e.g., exfoliating cleansers, toothpaste), while infant exposure may occur through breast milk, plastic feeding bottles, and mouthing behaviors with toys (Liu S. et al., 2023). In conclusion, humans are ubiquitously exposed to MPs through diverse routes, and internal exposure is confirmed by detection in various human tissues and biofluids, highlighting a significant exposure burden. Relevant studies on human MPs exposure are summarized in Table 1.

**TABLE 1:** Overview of the literature investigating microplastics (MPs) exposure in different samples of the human body.

Sample type	Detection technology	Most common polymer	Content	Shapes and sizes	References
Stomachs of 26 cadavers	μ-Raman	PE (30.5%), PP (13.9%), PMMA, (13.9%)	9.4 ± 10.4 particles/per individual	Fiber (52.04%): 1,196.6 ± 907.1 μm; fragments (39.80%): 330.4 ± 261.4 μm. blue (38.8%); black (24.5%)	Ozsoy et al., 2024
Heart from 15 cardiac surgery patients	Laser direct infrared; scanning electron microscopy	PET (77%), PU (12%)	–	Threads and rods; 20 ∼ 100 μm	Yang Y. et al., 2023
Lung samples from histological lung cancer or lung volume reduction surgery	Fourier transforminfrared micro-spectroscopy	PP (23%), PET (18%), resin (15%)	0.91 ± 0.95 particles/g of tissue (male); 0.33 ± 0.52 particles/g of tissue (female)	Fibre (49%), fragment (43%), or film (8%). Mean length: 223.10 ± 436.16 μm; width: 22.21 ± 20.32 μm	Jenner et al., 2022
Liver specimens of patients with chronic liver disease	Fluorescence microscopy; μ-Raman	PS, PVC, PET, PMMA, POM and PP	3.2∼9.9 MPs/g tissue (patients with cirrhosis); 0.0–1.5 particles/g in normal tissue	3.0 ∼ 29.5 μm (median is 9.8 μm)	Horvatits et al., 2022
20 samples of amniotic fluid and placenta from 10 preterm patients	Stereomicroscope; fourier transforminfrared micro-spectroscopy	CPE and calcium zinc PVC Stabilizer	Amniotic fluid: 0–8 particles; placenta: 0–10 particles	10 ∼ 50 μm	Halfar et al., 2023
Female uterine fibroids tissues and myometrium samples	Raman spectroscopy	PE (31.4%), PP (18.1%) and PE-co-PP (15.2%) in diseased tissue; PE (30.2%), PP (20.9%) and PE-co-PP (18.6%) in normal tissue	2.5 ± 1.66 (patient) and 1.05 ± 0.92 (control) MPs /g of tissue	Fiber (46.2%) and debris (30.8%). Brown (34.6%), blue (30.8%) and grey (19.2%). Mean length: 15.14 μm; width: 10.26 μm	Xu et al., 2024
Semen samples from ten healthy young men	μ-Raman	PP, PS, PE and PET	A total of 16 MPs were detected in six samples	Irregular fragment. 2 ∼ 6 μm. Blue, orange	Montano et al., 2023
Feces samples from 26 young male students	Fourier transforminfrared micro-spectroscopy	PP (61.0%), PET (17.2%), PS (3.4%)	95.8% participants tested positive for MPs. 1 ∼ 36 particles/g; 0.01 ∼ 14.6 mg/participant	20 ∼ 800 μm	Zhang et al., 2021

PE, polyethylene; PP, polypropylene; PVC, polyvinyl chloride; PS, polystyrene; PMMA, polymethyl methacrylate; PET, polyethylene terephthalate; PU, polyurethane; POM, polyformaldehyde; CPE, chlorinated polyethylene.

## 3 Mechanistic investigation into the impact of MPs on neurobehavioral disorders

Microplastics bridge environmental exposure and nervous system effects by accessing the central nervous system (CNS) via multiple pathways that either traverse or bypass the BBB, as supported by experimental animal and engineered BBB studies. Notably, *in vivo* oral exposure to PS-NPs induces transendothelial transcytosis, leading to their accumulation in the mouse brain (Sun and Song, 2025), while *in vitro* studies show submicron (0.2 μm) PS particles increase endothelial permeability in a size-dependent manner (0.2 > 1.0 μm) via tight-junction impairment, facilitating paracellular leakage (Araujo et al., 2025). Additionally, biomolecular corona composition modulates passage—cholesterol-rich coronas promote membrane insertion and brain uptake, while protein coronas inhibit it—providing a mechanistic basis for differential access (Bai et al., 2024). Further, non-vascular routes (e.g., olfactory epithelium to olfactory bulb) and trophic transfer through the food chain enable direct neuronal/perineural access alongside bloodborne entry, validated in fish models (Liu et al., 2024; Urani et al., 2024).

### 3.1 The absorption and dissemination of MPs within the nervous system

Microplastics and NPs, recognized as emergent environmental contaminants, have been shown to exert toxic effects on neural cells. In a mechanistic study using primary cortical cells from neonatal Wistar rats (Adamiak et al., 2025), 25 nm PS-NPs were demonstrated to penetrate the BBB and enter the CNS. Analysis revealed that astrocytes internalized PS-NPs primarily via actin polymer-dependent phagocytosis, whereas neurons predominantly utilized endocytic pathways for uptake. Notably, the uptake efficiency of PS-NPs in astrocytes was found to be 4–6 times higher than that in neurons. In addition, passive penetration is also an important way for MPs/NPs to break through the BBB. For example, PS-MPs have been observed to downregulate the expression of tight junction proteins, such as Occludin and ZO-1, in brain tissue, which facilitates their transport into endothelial cells, disrupts the integrity of the BBB, and ultimately allows access to neurons (Ma et al., 2024). Furthermore, *in vivo* exposure to PS has been shown to increase the expression of apoptotic proteins such as BAX, Caspase 8, and Caspase 3 in the cerebellar tissue (Yin et al., 2022). In entomological studies, PS-MPs measuring 1–5 μm have been documented to penetrate the BBB and reach the brain regions of honeybees within 3 days following oral exposure (Pasquini et al., 2024). Critically, evidence from human studies corroborates these experimental findings. In a clinical case-control study of 28 patients, Xie et al. (2024) employed Py-GC/MS and LDIR to analyze cerebrospinal fluid, revealing that the BBB selectively permits the entry of specific MPs, including PS, PE, PP, and PVC. Their analysis specifically identified that PP and PE concentrations correlated with BBB permeability, but not with inflammatory markers.

Upon entering biological fluids (e.g., blood, cerebrospinal fluid), MPs/NPs rapidly adsorb biomolecules including proteins and lipids, forming a distinctive “biomolecular corona.” This corona enables MPs/NPs to traverse cellular membranes through passive diffusion, mediated by specific binding interactions between the adsorbed biomolecular corona and membrane components (Casella and Ballaz, 2024; Shannahan, 2017). By modulating properties such as hydrophilicity/hydrophobicity, surface charge, and aggregation state, the biomolecular corona critically determines the capacity of MPs to cross physiological barriers—including the BBB and placental barrier. Specifically, corona proteins can mediate MPs binding to specific receptors on brain capillary endothelial cells, initiating receptor-mediated endocytosis and facilitating active transport into brain tissue (Kobos and Shannahan, 2020). The hydrophobic characteristics conferred by the corona are governed by MPs/NPs surface properties such as ligand saturation, elastic modulus, and curvature parameters. Following cellular internalization, the liberated MPs/NPs may subsequently induce formation of novel protein aggregates through their corona constituents, thereby propagating intracellular damage (Han et al., 2024; Hua and Wang, 2022).

**FIGURE 2:**
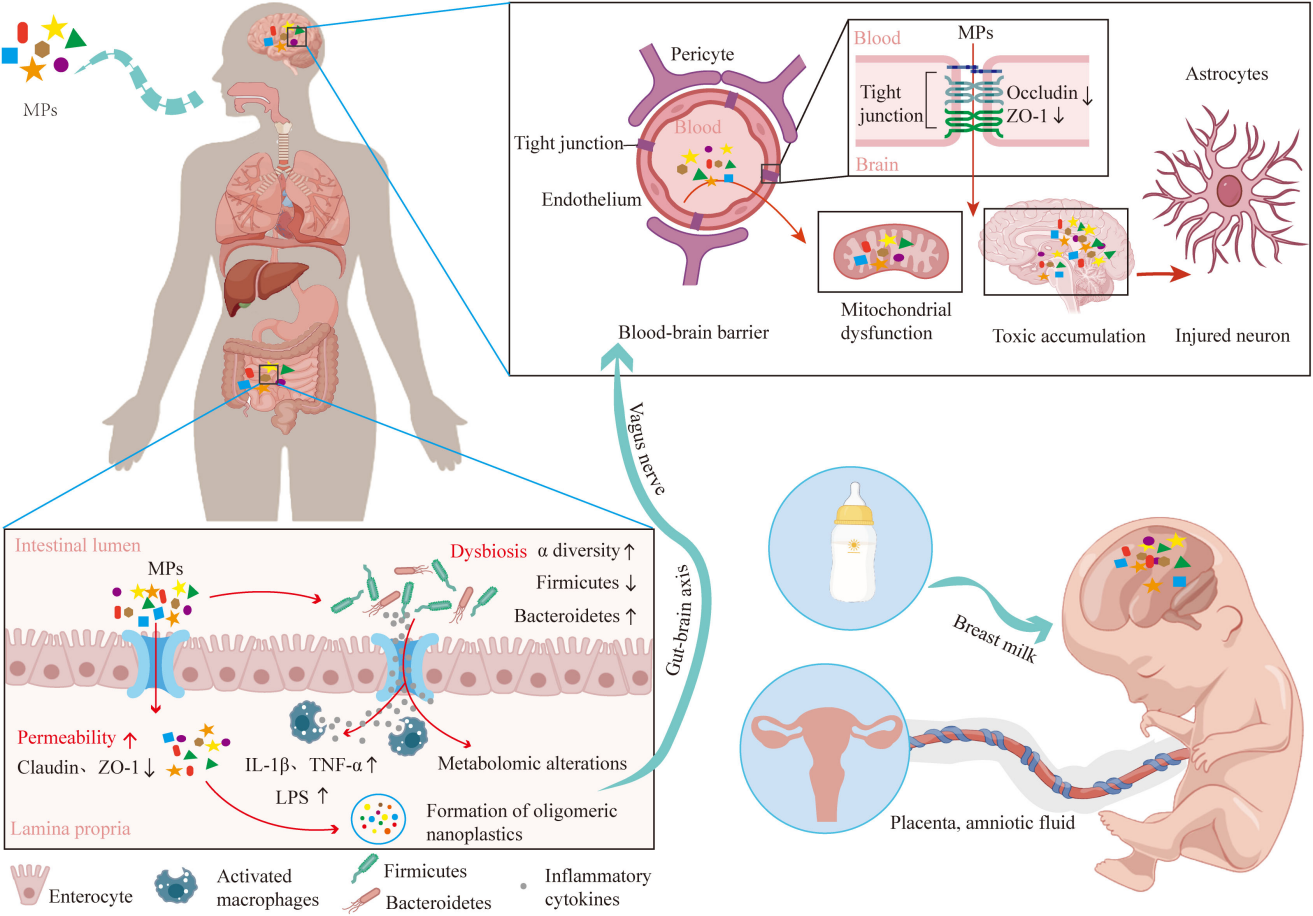
Schematic diagram of the translocation pathway of microplastics (MPs)/ nanoplastics (NPs) to the brain.

The gut-brain axis represents another critical pathway for MPs-mediated neurotoxicity. MPs may exert neurotoxic effects by traversing the gut-brain axis through various molecular mechanisms, including the disruption of gut microbiota homeostasis, impairment of intestinal barrier integrity, and direct interactions with the CNS. MPs can induce dysbiosis within the gut microbiota, which subsequently influences brain function (Shi et al., 2024; Sun H. et al., 2024). Specifically, animal studies show MPs can disrupt the α-diversity of gut microbiota and decrease the Firmicutes-to-Bacteroidetes (F/B) ratio, resulting in an increased relative abundance of pathogenic bacteria in ICR mice (Hu et al., 2023). Elevated levels of α-diversity have been associated with impaired emotional regulation and have been observed in adults diagnosed with major depressive disorder and schizophrenia (Gao et al., 2019; Jiang et al., 2015). Furthermore, inflammatory cytokines and metabolites produced as a consequence of gut microbiota imbalance can be transmitted to the brain via the vagus nerve, instigating oxidative stress and neuroinflammation (Wang et al., 2002), which are critical factors in the pathogenesis of neurodegenerative diseases. For instance, exposure to PS-MPs has been shown to significantly alter gut flora composition, correlating with decreased oxytocin levels in the medial prefrontal cortex (mPFC) and impaired social behaviors in C57BL/6 murine models (Wang L. et al., 2024). Moreover, MPs compromise the integrity of the intestinal barrier, increasing permeability and permitting harmful substances to enter systemic circulation. This disruption facilitates the transfer of inflammatory mediators, such as lipopolysaccharide (LPS), IL-1β, and TNF-α, to the brain, thereby exacerbating neuroinflammation (Jiang et al., 2023; Luan et al., 2024). The integrity of the intestinal barrier is crucial not only for regulating metabolic and inflammatory responses but also for cognitive and memory processes (Fan and Pedersen, 2021). Additionally, oligomeric NPs formed from the degradation of MPs enhance neurotoxicity and further impact cognitive functions. For example, in the male C57BL/6 J mice model, polylactic acid (PLA) MPs degrade in the gastrointestinal tract into oligomeric nanoparticles, which exhibit increased bioavailability and toxicity, thereby exacerbating neurotoxic effects in the brain (Liang B. et al., 2024). Consequently, the gut-brain axis serves as a pivotal pathway linking gut dysbiosis to brain health (see Figure 2).

Particle size and surface properties are key determinants of MPs neurotoxicity. Empirical studies have demonstrated that smaller particles, especially nanosized ones, exhibit enhanced efficiency in crossing the BBB compared to larger MPs, leading to brain accumulation and potential neurotoxicity. *In vivo* and *in vitro* evidence indicates submicron/nanoparticles (e.g., 0.293 and 0.2 μm) penetrate the BBB more rapidly than larger microparticles (Araujo et al., 2025; Bai et al., 2024). This is mechanistically explained by their higher surface area, distinct protein corona formation, and preferential uptake via endocytic pathways like clathrin- or caveolae-mediated endocytosis, which alter intracellular fate and reactivity (Bai et al., 2024; Han et al., 2024). For instance, research using 3D human BBB models indicates that particles measuring 0.2 μm PS-MPs demonstrate greater absorption and transendothelial transport across the BBB than 1.0 μm particles (Cho et al., 2024). Similarly, in developmental models using Sprague-Dawley (SD) rats, 25 nm PS-NPs infiltrated offspring brain regions, particularly the cerebellum, hippocampus, and prefrontal cortex more effectively than 50 nm particles via endocytosis (Zhang et al., 2024). Surface chemical modifications further modulates cellular uptake. *In vitro* studies using human brain microvascular endothelial cell line hCMEC/D3 show that carboxylated PS primarily utilize clathrin-mediated endocytosis, while aminated variants diffuses passively (Ma et al., 2024). Internalized PS-NPs preferentially target mitochondria, inducing dysfunction and neural damage (Tao et al., 2024). These relationships are summarized in Table 2.

**TABLE 2:** Relationship between microplastic characteristics and neurotoxic effects.

Species	Type	Size	Method of administration	Exposure dose	Neurotoxic effects and possible mechanisms	References
Male C57BL/6	PS	0.1, 5, and 50 μm	Oral gavage	10 mg/L	0.1 μm PS-MPs had the greatest effect. MPs can induce macrophage reduction, thereby affecting the physical and mental health by modulating the microbiota–gut–brain axis.	Kuai et al., 2024
C57BL/6⋅J mice	PS, PS-COOH, and PS-NH2	80 nm	Intranasal administration (INA) exposure	2.5 mg/kg BW (low), 5 mg/kg BW (medium) and 10 mg/kg BW (high)	PS-NH2 exhibited the greatest accumulation in the mice brain after exposure for 7 days. After the mice were exposed to PS-NH2 by INA means for 28 days, the exploratory ability and spatial learning ability were obviously damaged in a dose-dependent manner.	Sun M. et al., 2024
Male BALB/c mice	PS	0.5, 4, and 10 μm	Oral gavage	100 μg/L and 1,000 μg/L for 180 consecutive days	There was a concentration-dependent trend, but no particle size-dependent differences were seen in the neurotoxicity of MPs. PS-MPs disrupt the blood-brain barrier and cause hippocampal inflammatory responses, leading to cognitive and memory deficits	Jin et al., 2022
Male Wistar rats	LDPE	<30 μm	Oral gavage	10 mg/kg BW per day for 3 and 6 weeks.	BBB permeability increased significantly in both 3-and 6-week MP treatment groups. Longer LDPE-MP exposure led to progressively worse BBB dysfunction, oxidative stress, and neuronal injury in rats.	Forutan et al., 2025
Danio rerio	PS	44 nm	Exposure solution	1, 10, and 100 μg/L for 30 days	3,4-dihydroxyphenylacetic acid (DOPAC) was decreased in a dose-dependent manner.	Teng et al., 2022
Zebrafish larvae	Virgin and aged PS-MPs	1 μm	Glass petri dishes containing 30 mL of exposure solution per well.	0.1–100 μg/L	UV-aged 1 μm PS-MPs caused stronger neurotransmitter disturbances than virgin MPs.	Xiang et al., 2023
Zebrafish	Virgin and photoaged PS	10 μm	Culture dishes	0, 0.1, 1, 10, and 100 μg/L.	Photoaged MPs induced more oxidative stress and neurotransmitter imbalance than virgin MPs at similar doses, resulting in more severe neurotoxicity.	Ding et al., 2023
Zebrafish embryos	PS	500 nm	Exposure solution	0.1, 1 and 10 ppm for 6 days	Exposure to PS-MPs reduced locomotor activity at 1 ppm followed by 0.1 and 10 ppm.	Suman et al., 2023
Zebrafish larvae	PS	50, 100 nm	Larvae were placed in Embryonic media containing PS-NPs for a period of 96 h	1,000 ppm	50 nm PS-NPs have higher BBB penetration compared with 100 nm PS-NPs. Unlike 100 nm PS-NPs, 50 nm PS-NPs accumulated in zebrafish larval brains, causing behavioral abnormalities, EEG changes, dopaminergic disruption, and increased anxiety, which were absent with 100 nm particles.	Hwang et al., 2022
Zebrafish	PE	40–47 μm	Waterborne route and the foodborne (microplastic-contaminated daphnids) route	0.1, 1, 10 mg/L	Zebrafish exposed to 40–47 μm polyethylene MPs via water or food showed distinct neurotoxic pathways; foodborne exposure caused more behavioral hyperactivity. Highest concentration (10 mg/L) ingested more microplastics than other treatments.	Yu et al., 2022
Zebrafish	PS	70 nm	Glass tanks containing test solutions	0.5 and 1.5 ppm for seven consecutive days	70 nm PS-NPs accumulated in zebrafish brains, altering aggression, shoaling, and circadian activity in a concentration-dependent manner.	Sarasamma et al., 2020
Zebrafish	PLA	1–30 μm	Exposure solution	1, 10, and 20 mg/L	PLA concentration-dependent inhibition of the development of neurons and lateral mounds in zebrafish larvae leads to reduced exploration behavior, poor memory, abnormal behavior, anxiety and neuronal loss in zebrafish larvae.	Qian et al., 2025

PE, polyethylene; PS, polystyrene; PLA, polylactic acid; LDPE, low-density polyethylene.

Upon entering the CNS, MPs accumulate in specific brain regions and cell types, correlating with structural and functional impairments. Studies suggest that MPs can accumulate in cognitive-related brain regions such as the hippocampus and cortex, with reported correlations to neuronal structual alterations and limbic system changes. Exposure to PS-MPs/NPs have been associated with reduced pyramidal cells and dendritic spines in the hippocampus, along with DNA damage in prefrontal cortical neurons (Kaur et al., 2024; Ma et al., 2024). Neurotoxic potential may depend on particle localization—inside neural cells or extracellular neuropil—and properties such as size. Internalized particles have been observed in astrocytes and neural stem cells, accompanied by decreased viability and transcriptional shifts (Liu et al., 2024; Marcellus et al., 2024). Neuronal uptake of PS-NPs has also been reported, with intracellular trafficking and possible protein interactions linked to degenerative phenotypes (Han et al., 2024; Shan et al., 2022). Animal studies further indicate parenchymal accumulation and microglial activation, where intracellular 50–500 nm particles tend to correlate with cytotoxic effects (Bai et al., 2024).

### 3.2 Oxidative stress and neuroinflammation

Oxidative stress is a central mechanism in MPs-induced neurotoxicity. Exposure to MPs has been shown to induce the production of reactive oxygen species (ROS) in neuronal cells, which subsequently leads to a reduction in catalase activity and glutathione levels. This oxidative stress can result in damage to DNA, proteins, and lipids, ultimately compromising neuronal integrity (Marcellus et al., 2024; Vojnits et al., 2024). For example, in zebrafish models, PS-NPs have been associated with elevated levels of ROS, leading to alterations in locomotor and behavioral patterns (Sarasamma et al., 2020). *In vitro*, in the human neuroblastoma cell line SH-SY5Y, exposure to PS-NPs has been linked to an increase in mitochondrial superoxide (mitoSOX) and mitochondrial ROS (mROS) production (Ma et al., 2024). The oxidative stress-induced lipid peroxide is a significant mechanism in the pathogenesis of depression, with the accumulation of lipid peroxidation (LPO) contributing to neuronal cell death and dysfunction (Liu et al., 2024; Prust et al., 2020). Lipid peroxidation generates harmful by-products, such as malondialdehyde and 4-hydroxynonenal, which can modify proteins and disrupt cellular functions (Allowitz et al., 2024). These lipid-derived aldehydes may also function as secondary signaling molecules, influencing metabolic pathways and contributing to neurodegeneration (Perluigi et al., 2024).

Oxidative stress can result in the accumulation of free radicals and inflammatory responses, which can damage neuronal cells and contribute to neurodegenerative diseases (Singh et al., 2024). *In vivo*, in the hippocampal tissues of mice exposed to PS-MPs, there was a significant increase in the mRNA expression levels of inflammation-related genes (*Tnf-a*, *Il-1b*, *Il-6*, *Cxcl10*, and *Mcp-1*), indicating heightened inflammatory responses (Cho et al., 2024). *In vitro* studies indicate PS has the capacity to bind to and activate BV2 microglial cells, thereby inducing pro-inflammatory reactions and activating inflammatory pathways, such as the NF-κB pathway, which results in the production of pro-inflammatory cytokines (Li et al., 2024). Chronic activation of microglia is a critical factor in the progression of neurodegenerative diseases (Gettings et al., 2024), and this persistent neuroinflammation can compromise the integrity of the BBB, increasing neuronal cells’ susceptibility to external harmful substances, which can lead to neuronal dysfunction, damage, and loss, ultimately impacting mental health and manifesting as symptoms such as depression and anxiety (Buzenchi Proca et al., 2024). Thus, both *in vitro* and *in vivo* evidence converge to highlight oxidative stress and subsequent neuroinflammation as pivotal events in MPs-induced neurotoxicity.

### 3.3 Endocrine disruption

Endocrine disruption is another consequence of microplastic exposure. The chemical additives present in MPs, including plasticizers and antioxidants, have the potential to disrupt the normal functioning of the neuroendocrine system, affecting hormone synthesis, secretion, and metabolism. MPs can leach endocrine-disrupting chemicals (EDCs), such as bisphenol A (BPA), phthalates [e.g., di(2-ethylhexyl) phthalate (DEHP), dibutyl phthalate (DBP)], and polyfluoroalkyl substances (PFASs) (Maradonna et al., 2022). Additionally, MPs can function as molecular sponges, absorbing and concentrating these EDCs within their structure. Notably, BPA and phthalates are classified as steroidogenic EDCs. These compounds can mimic hormones, bind to hormone receptors, and exert either agonistic or antagonistic effects, thereby disrupting steroidogenesis and interfering with neurogenesis, synaptic transmission, and brain sexual differentiation (Ahn and Jeung, 2023).

Dysregulation of the neuroendocrine system can adversely affect learning, memory and behavior, potentially leading to neurodegenerative diseases. For instance, imbalances within the neuroendocrine system, particularly involving the hypothalamic-pituitary-adrenal (HPA) axis, can severely disrupt hormonal balance, resulting in cognitive and behavioral impairments (Erlanger et al., 1999). Gonadal hormones, for example, modulate cholinergic pathways and influence neurotransmitter systems, which in turn can interfere with cognitive function and mood regulation (Schipper, 2016). Studies indicate that 1.5 ppm of PS-NPs can induce elevated cortisol levels in zebrafish (Sarasamma et al., 2020). These heightened corticosteroid levels may trigger abnormal microglial and astrocytic responses, further exacerbating neuroinflammation and impairing cognitive function and neuroanatomy, particularly in the hippocampus, which is crucial for learning and memory (Nesan and Kurrasch, 2020; Scassellati et al., 2021).

### 3.4 Impaired cellular housekeeping

Microplastics /NPs commonly trigger autophagy initiation markers in neuronal models, yet they frequently impair autophagic flux and lysosomal degradation, leading to a dissociation between autophagosome formation and cargo clearance. Multiple studies report elevated levels of LC3, p62, and Atg proteins, accompanied by reduced colocalization with lysosomal markers or delayed clearance of protein aggregates (Casella and Ballaz, 2024; Nie et al., 2021). Although direct neuron-specific evidence that PS-NPs inhibit the ubiquitin–proteasome system (UPS) remains limited, studies on related nanoparticles indicate altered ubiquitination and proteostasis. For instance, silica-coated magnetic nanoparticles suppressed proteasome gene expression and activity, while promoting cytoplasmic inclusion formation in SH-SY5Y cells and primary neurons in a dose-dependent manner (Liu et al., 2024). Moreover, mechanistic studies suggest that PS-NPs can modify ubiquitin structure and ubiquitination patterns, potentially disrupting UPS-mediated protein quality control (Nie et al., 2021). Given that neuronal proteostasis relies on both autophagy and the UPS, nanoplastic-induced lysosomal dysfunction and delayed aggregate degradation—such as slowed α-synuclein clearance—may indirectly increase UPS burden and promote inclusion formation, even without direct evidence of proteasome inhibition (Nie et al., 2021).

Substantial evidence supports mitochondrial dysfunction as a key mechanism in MPs/NPs neurotoxicity. Multiple studies document mitochondrial accumulation of PS-NPs, loss of membrane potential, ATP depletion, and impaired respiration—particularly through Complex I interference. Molecular docking and dynamics simulations further support PS-NPs disruption of Complex I, providing a mechanistic basis for these deficits (Huang et al., 2023). These energetic deficits activate AMPK/ULK1 signaling, driving excessive mitophagy; inhibition of AMPK or autophagy reduced mitophagy and preserved cell viability, confirming the causal role of this pathway. In addition, mitochondrial damage can trigger intrinsic apoptosis, characterized by outer membrane permeabilization, cytochrome c release, and caspase-3 activation (Dal Yöntem and Aydoğan Ahbab, 2024).

Although Endoplasmic reticulum (ER) stress and the unfolded protein response (UPR) are noted in broader nanoplastic reviews, direct and detailed studies in PS-NPs-exposed neurons remain limited. Available literature positions ER stress, oxidative stress, and proteostasis disruption as interconnected contributors to neuronal homeostasis collapse. For example, ER stress can exacerbate oxidative damage and impair protein folding, thereby increasing demands on autophagy and the UPS, while mitochondrial dysfunction depletes ATP essential for these processes (Huang et al., 2023; Nie et al., 2021). Finally, NPs have been associated with multiple cell death pathways, including ferroptosis (via p53-mediated ferritinophagy and GPX4 depletion), pyroptosis (via TSC2/TFEB-related lysosomal defects), and intrinsic apoptosis, indicating context-dependent terminal outcomes for neuronal homeostasis (Dal Yöntem and Aydoğan Ahbab, 2024; Huang et al., 2023; Liu Z. et al., 2023).

### 3.5 Changes in neurotransmitter and synaptic function

Understanding how MPs and NPs affect neurotransmission requires reconciling limited and often conflicting evidence, which currently points to a highly context-dependent nature of neurotoxicity.

Studies indicate that exposure to PS-NPs significantly downregulates dopamine levels in zebrafish (Ren et al., 2024), mechanistically linked to MPs-triggered neuroinflammation that inhibits tyrosine hydroxylase, a key enzyme in dopamine synthesis (Wang et al., 2025). Conversely, ultraviolet rays-aged microplastics increase dopamine in zebrafish (Xiang et al., 2023), highlighting how physicochemical alterations impact neurotoxicity. The serotonergic system shows similar contingency, where the effect of MPs on serotonin (5-HT) depends on whether the exposure occurs in isolation or in a mixture (Ding et al., 2023; Ren et al., 2024). Regarding the cholinergic system, exposure to PS- and PP-MPs has been shown to reduce the activity of acetylcholinesterase (AChE), a critical enzyme for nerve impulse transmission and a recognized biomarker of neurotoxicity (Zhang et al., 2025). This inhibition can lead to alterations in learning, memory, and anxiety-like behaviors in mice (Wang J. et al., 2024; Yang G. et al., 2023). Additionally, oxidative stress and mitochondrial dysfunction induced by MPs are believed to impair GABAergic neuron function, leading to decreased GABA levels in the cerebral cortex of rats exposed to PS-MPs (Oyovwi et al., 2024), with implications for epilepsy and ASD (Bruzelius et al., 2021). In summary, the question of how MPs/NPs disrupt neurotransmission cannot be answered by a single mechanism but must be framed by the interplay between particle properties, environmental transformations, and biological context.

Moreover, exposure to MPs can impair neuronal synaptic function, which is essential for the normal operation of neural networks. Synapses are critical structures for information transmission between neurons, and synaptic dysfunction can lead to neurological behavioral changes, such as deficits in learning and emotional disturbances. For instance, PS-MPs have been shown to reduce dendritic spines in the hippocampal corpus callosum-1 region, which is closely associated with learning and memory, and decrease the expression levels of Syt 1 and Bdnf mRNA, which are involved in neuronal development and synaptogenesis in hippocampal tissue (Jin et al., 2022). Exposure to PS-NPs has been found to cause brain damage in mice, activate astrocytes and microglia, induce cytoplasmic vacuolization of neurons, and result in the thinning of cell layers in the hippocampus and cortex, as well as abnormalities in pyramidal cells. These changes adversely affect synaptic function and induce anxiety- and depression-like behavioral alterations (Ma et al., 2024). Furthermore, PS-MPs can modify the morphology of dendrites in prefrontal cortex neurons of mice by influencing neurodevelopment-related signaling pathways, leading to reduced dendritic branching and decreased dendritic spine density (Suman et al., 2024). Such alterations may impact neuronal connectivity and information transmission, thereby affecting cognitive and behavioral functions.

### 3.6 Gene expression and neural networks

Exposure to MPs will influence gene expression in neural cells and disrupt associated signaling pathways. In mouse models, low-density polyethylene (LDPE) or oxidized low-density polyethylene (Ox-LDPE) has been shown to disrupt cholinergic signaling pathways in the cerebral cortex and hippocampus, impairing the expression of choline acetyltransferase (ChAT), SLC5A7, and vesicular acetylcholine transporter *Slc18a3* (VAChT). This disruption leads to blocked acetylcholine synthesis and secretion, resulting in cognitive impairments and affecting mood and memory in C57BL/6 mice (Wang L. et al., 2024). Exposure to PS-NPs has been associated with changes in gene expression in rat brains, particularly those related to neurological function and cell death, including the upregulation of apoptosis-related genes (e.g., *Bax*, *p53*, *Bcl-2*) and inflammatory factors (e.g., *TNF*-α, *IL-6*, *NF*-κ*B*) (Wang H. et al., 2024). RNA-seq analysis has revealed significant alterations in the gene expression profile of astrocytes following 7 days of microplastic exposure, with upregulated pathways related to inflammation, immune response, migration, proliferation, and endoplasmic reticulum stress, while pathways related to lipid metabolism were downregulated (Marcellus et al., 2024). Furthermore, exposure of pregnant mice to plastic particles of varying sizes and surface modifications has demonstrated that NPs can alter gene expression in the fetal thalamus, leading to oxidative damage and neuronal apoptosis. The differentially expressed genes were primarily enriched in pathways related to oxidative phosphorylation and GABA synapses, resulting in reduced GABA neurotransmitter levels and anxiety-like behaviors in adult offspring (Yang et al., 2022). This suggests that MPs may induce neuronal apoptosis and inflammatory responses by modulating gene expression, thereby impairing cognitive function.

In addition to affecting cellular biocompatibility and signal transduction, exposure to MPs may also influence neuroplasticity through immune response modulation. Studies indicate that MPs can activate the immune system, leading to chronic inflammatory responses that may impair neuronal plasticity and function (Ojha et al., 2024). Within the nervous system, the activation of immune cells such as microglia and astrocytes triggers the release of various cytokines, which not only participate in immune responses but also influence neuronal growth and regeneration (Digiovanni et al., 2022). For instance, the presence of MPs may alter microglial function, disrupting synaptic plasticity in neurons and negatively impacting cognitive functions such as learning and memory (Zhao et al., 2023). Additionally, MPs may indirectly affect neuroplasticity through immune regulation in the gut. NPs have been shown to activate intestinal macrophages, which release interleukin-1 (IL-1)—a cytokine that influences brain immunity—resulting in microglial activation and cognitive decline (Sofield et al., 2024; Yang Q. et al., 2023). Therefore, the relationship between MPs-induced immune modulation and neuroplasticity warrants further investigation to elucidate their potential implications for neurological health.

## 4 Neurobehavioral disorders induced by MPs

The neurotoxic mechanisms of MPs culminate in functional deficits, manifesting as various neurobehavioral disorders in experimental models, with growing concern for human relevance.

### 4.1 Anxiety and depression

Substantial evidence from animal models links MPs exposure to the development of anxiety- and depression-like behaviors. The impact of MPs on emotional cognition in animals, particularly concerning behavioral modifications, reveals substantial disruption to the nervous system. *In vivo* studies using zebrafish, which exhibit a 70% genetic homology with humans, serve as an effective model for such studies (Gupta et al., 2023). Following exposure to PS-NPs, zebrafish demonstrated notable behavioral alterations, including diminished locomotor activity, heightened aggression, reduced social grouping, and impaired predator avoidance (Jewett et al., 2022). These findings underscore the pervasive and significant effects of MPs on animal emotional states. In mammalian studies, exposure to PS-NPs has been linked to a marked reduction in social behaviors and the emergence of anxiety- and depression-like symptoms in murine models (Chen et al., 2023). The observed anxiety-like behaviors in murine models following PS-NPs exposure are mechanistically supported by the previously documented activation of the HRAS-derived Perk-NF-κB inflammatory pathway in the brain (Li et al., 2024). Meanwhile, maternal exposure to PS-MPs has been shown to affect the social behaviors of offspring adversely, resulting in decreased engagement in social interactions and increased anxiety (Kaur et al., 2024). The duration of exposure to MPs appears to exacerbate the neurobehavioral changes observed (Jin et al., 2022; Wang J. et al., 2024). Furthermore, the reduced dendritic spine density in the prefrontal cortex (Suman et al., 2024) provides a structural basis for the reported cognitive and emotional deficits, creating a direct link from cellular pathology to behavioral phenotype.

Microplastics also influence mood-regulating regions of the brain. Notable reductions in dendritic length and spine density in the PFC have been documented following exposure to PS-MPs, leading to compromised neuronal connectivity (Suman et al., 2024). Damage to the PFC, a region essential for executive functioning and emotional regulation, may precipitate behavioral changes and cognitive deficits (Belujon and Grace, 2008). These alterations are associated with decreased expression of brain-derived neurotrophic factor (BDNF), a critical molecule for neuronal health and functionality (Suman et al., 2024). Additionally, exposure to PS-MPs has been linked to a reduction in neuronal cell populations within the hippocampal dentate gyrus and hippocampal horn, potentially impairing memory and emotional responses. Evidence of neuronal damage has also been observed in the amygdala and hypothalamus, further influencing emotional regulation (Kaur et al., 2024). Collectively, these findings from animal models suggest that MPs may disrupt emotional regulation by impairing neuronal structure and function in key brain regions, thereby contributing to neurobehavioral disorders such as anxiety and depression.

### 4.2 Cognitive impairment

Microplastics exposure is consistently associated with cognitive deficits across diverse experimental models. Animal studies have demonstrated that MPs disrupt learning and memory processes. In murine experiments, exposure to PS-MPs resulted in neurobehavioral changes characterized by increased latency in reaching a platform and impaired spatial learning and cognitive function, as evidenced by the water maze test. This impairment is associated with DNA damage in neuronal cells within the mPFC (Kaur et al., 2024). In zebrafish models, prolonged exposure to polyglycolic acid (PGA) has been shown to alter 5-HT levels in the brain via the gut-liver-brain axis, significantly diminishing motor performance and inducing cognitive deficits (Luan et al., 2024; Vegas-Suarez et al., 2022). Traditional animal experiments have often employed gavage methods to simulate microplastic exposure; however, air pollution represents a significant pathway for human exposure. A novel air exposure model in C57BL/6 mice, utilizing endotracheal drip, revealed that inhaled MPs promote M1 polarization of microglia through the lung-brain axis, subsequently leading to cognitive impairments in mice (Kang et al., 2024). Furthermore, a clinical cohort study involving 80 university students confirmed that reduced sleep exposure to MPs significantly enhance cognitive performance, attention, and memory, primarily by improving sleep oxygen saturation and neurotransmitter metabolism (Yang et al., 2024). These findings suggest that MPs may disrupt normal neuronal function, impairing neural signaling and neurotransmitter homeostasis, which in turn affects learning and memory capabilities. Cognitive decline may also be influenced by dysregulated competing endogenous RNAs (ceRNAs), with significant dysregulation of 96 mRNAs associated with synaptic dysfunction observed in the mouse prefrontal cortex (Chu et al., 2022).

Epidemiological studies and research on neurodegenerative hallmarks provide tentative links to human disease. Epidemiological studies have reported a significant increase in the frequency of micronuclei (MN), nucleoplasmic bridge (NPB), and nucleoblast bud (NBUD) formation in human peripheral blood lymphocytes following exposure to PE-MPs (Cobanoglu et al., 2021). The elevation of these metrics has been linked to neurodegenerative disorders, including Alzheimer’s disease and Parkinson’s disease (Migliore et al., 2011), providing new insights into the mechanisms underlying cognitive decline. Notably, the concentration of MPs in brain samples from dementia patients was found to be significantly higher than in normal brain samples (Nihart et al., 2025). In animal studies, exposure to PS-MPs and NPs resulted in neurobehavioral deficits in C57BL/6 J mice, including reduced mobility, grip strength, and coordination. Single-cell nuclear transcriptomics studies of the brain suggest that NPs may induce Parkinson’s disease-like neurodegenerative lesions by disrupting mitochondrial energy metabolism and adenosine triphosphate (ATP) production in substantia nigra and striatal excitatory neurons (Liang et al., 2022). Additionally, PS-NPs have been shown to induce Parkinson’s-like symptoms in the invertebrate Caenorhabditis elegans (Youssef et al., 2021) and exacerbate α-synuclein aggregation in SH-SY5Y cells derived from human neuroblastoma, a hallmark of Parkinson’s disease (Jeong et al., 2024; Polinski et al., 2018). In zebrafish models, MPs exposure may induce oxidative stress in the brain, leading to neuroinflammation and neurotoxicity, which could be associated with the development of schizophrenia and alterations in behavioral patterns (Savuca et al., 2024). These findings suggest a close association between MPs exposure and the onset of cognitive disorders such as neurodegenerative diseases. While direct human causation remains unproven, these converging lines of evidence from *in vitro*, animal, and preliminary human studies suggest a disconcerting potential link between MPs exposure and cognitive disorders, including neurodegenerative diseases.

### 4.3 ASD and ADHD

Recent research has indicated a potential link between prenatal and early postnatal exposure to MPs and the subsequent development of ASD and ADHD-like phenotypes in animal models. Zaheer et al. (2022) demonstrated that prenatal exposure to PE in C57BL/6J mice leads to characteristics similar to autism spectrum disorder, and results in impaired social interaction and repetitive behaviors in mouse models. Dysbiosis of the microbiota and elevated levels of *EGR-1* and *ACR* genes may be evidence of ASD-like characteristics after PE exposure. Furthermore, studies *in vivo* involving zebrafish have shown that early exposure to MPs leads to significant demethylation of DNA in adult stages, with an upregulation of genes associated with neurotoxicity, such as *slc6a4b* and *oxtrl*. These genetic alterations may contribute to hyperactivity in zebrafish (Im et al., 2022), a behavioral characteristic that parallels one of the clinical manifestations associated with ADHD. In recent studies, long-term exposure to 23 nm PS-NPs at a dose of 10 μg/day/kg throughout the entire life stage of wild-type C57BL/6 J mice disrupted the key developmental milestones of the offspring. Mice exposed to PS-NPs exhibited signs of ADHD during development (catwalk, olfactory preference) and in adulthood (increased entries in the Y-maze and three-chamber test), including increased risk-taking behavior and hyperactivity, as well as impaired motor learning and executive function. These behavioral impairments were associated with alterations in the expression of genes and synaptic proteins related to ADHD. Additionally, the increase in lipofuscin granules and lysosomal damage in neurons and microglia after lifelong exposure to nanoplastics indicated accelerated brain aging (Vignon et al., 2025).

In addition, MPs may act as carriers for endocrine disruptors and organic pollutants, thereby increasing the risk of neurotoxicity. These substances have been independently linked to neurodevelopmental disorders. For instance, nitrogen dioxide and polychlorinated biphenyls are directly correlated with an elevated risk of ASD (Duque-Cartagena et al., 2024), while lead and phthalates—common contaminants associated with MPs—have been identified as risk factors for ADHD (Chopra et al., 2014; Hong et al., 2015). When ingested by organisms, MPs can release adsorbed contaminants, potentially leading to a synergistic effect that results in cellular and organ damage. It is important to note, however, that the existing evidence primarily stems from correlational studies in humans and causative animal models, and definitive causal relationships in humans remain unclear (Nihart et al., 2025). For instance, the impaired clearance of microplastic-associated chemicals in children diagnosed with ASD or ADHD may be attributable to either the exposure itself or the disease state affecting metabolic processes (Stein et al., 2023). Future research should focus on longitudinal cohort studies combined with mechanistic experiments to systematically investigate the causal relationships between the dosage and timing of MPs exposure and neurobehavioral abnormalities.

### 4.4 Transgenerational effects of maternal exposure

A particularly concerning aspect of MPs toxicity is its potential for transgenerational effects, as evidenced by both animal models and preliminary human findings. Research indicates that maternal exposure to MPs in both animal models and humans has significant transgenerational effects. In animal studies, exposure to MPs during prenatal and lactational periods has been associated with impairments in neurodevelopment among offspring, leading to neurobehavioral abnormalities (He and Yin, 2024). Research indicates that pregnant rats exposed to PS-NPs can produce neurotoxicity in their offspring through a p53-mediated ferroptosis mechanism, ultimately resulting in cognitive and memory function impairments. Specifically, PS-NPs induce excessive production of ROS, activate the p53 signaling pathway, and subsequently initiate NCOA4-mediated ferritinophagy, resulting in iron overload, lipid peroxidation, and ferroptosis within the hippocampal tissue of offspring (Chen J. et al., 2024).

Research utilizing rodent models has shown that NPs and MPs can be transferred across the placenta and through lactation, leading to their accumulation in the brains of fetuses and neonates. This accumulation has been found to disrupt the levels of cerebral monoamine neurotransmitters and the signaling of amino acids in the hippocampus. Prenatal exposure to these substances results in cortical thinning and hyperproliferation in fetal rats, while not affecting neuronal differentiation. These alterations are associated with the emergence of anxiety-like behaviors and deficits in spatial memory during adolescence (Tian et al., 2024). Furthermore, disposable paper cups have been identified as a source of microplastic release when containing hot beverages, with tissue-specific deposition of MPs noted in fetuses and placentas. Such exposure has been linked to metabolic and immune dysfunction, thereby heightening the risks of neurodegenerative diseases and miscarriage in murine models (Chen et al., 2024). Additionally, PS-MPs can infiltrate mammary glands and breast milk in lactating rats, crossing the BBB to accumulate in the brains of offspring. This accumulation of PS is associated with the manifestation of anxiety- and depression-like behaviors, as well as diminished social interaction in offspring mice (So et al., 2023). Notably, prenatal exposure appears to yield more severe consequences compared to lactational exposure (Shin et al., 2023). Epidemiological data in humans also suggest that maternal exposure to NPs during pregnancy and lactation may predispose offspring to neurodevelopmental disorders (Tian et al., 2024). Moreover, exposure to MPs has been found to downregulate markers of mature neurons, thereby compromising neural activity in offspring (Hua et al., 2022). This evidence of transgenerational toxicity underscores the notion that the health risks associated with MPs extend beyond the individuals directly exposed, potentially inflicting long-term consequences on subsequent generations.

## 5 Recommendations and measures to reduce MPs pollution

To mitigate and manage microplastic pollution, it is essential to establish a coordinated framework aimed at minimizing exposure risks through a multi-faceted approach. Research indicates a positive correlation between the concentration of MPs in feces and the frequency of utilizing takeout packaging, with bottled water containing microplastic levels that can be three to five times higher than those found in tap water (Song et al., 2024; Zhang et al., 2023). At the individual level, it is advisable to prioritize the use of stainless steel or glass containers over disposable tableware and to encourage the combined utilization of community tap water systems alongside household water purification devices. For public infrastructure, the water supply infrastructure should progressively eliminate aging PE pipes in favor of food-grade stainless steel materials while also establishing a dynamic monitoring network for MPs in drinking water. Indoor environments exhibit significantly higher concentrations of MPs compared to outdoor settings. Under the influence of ultraviolet rays, the potential release of microplastics from materials such as decorative tiles, insulating foam wallpaper and sheet paper increases. In contrast, the average change in tiles and paints is relatively small. Among them, materials such as wallpaper and board products show a higher human impact index (Kang et al., 2025). Basic protective measures can be implemented through two daily ventilation sessions of 30 min each, supplemented by HEPA filter purification systems.

The management of microplastic pollution in public spaces necessitates a comprehensive control strategy. MPs generated from transportation and outdoor activities are highly dispersive and challenging to regulate. For instance, tire wear accounts for 28% of global marine microplastic pollution, with a single cross-country journey potentially releasing over 20,000 particles (Forster et al., 2023; Smyth et al., 2025). Municipalities should promote the use of rubber-modified asphalt for roadways and establish environmental standards for sports facilities such as the use of recycled polyester fiber in sportswear. The industrial sector should expedite the research and development of bio-based tires, with prototypes of dandelion rubber tires currently undergoing testing. In response to the pollution issue of events, environmental performance indicators can be incorporated into the event rating system.

The integration of technological innovation and institutional support is crucial. Advancements in microbial degradation technology must overcome existing efficiency limitations. For example, the genetically modified strain of *Ideonella sakaiensis* has demonstrated a twelvefold enhancement in the efficiency of polyethylene terephthalate (PET) degradation, and the resultant byproducts are amenable to conversion into biofuels (Chen et al., 2020; Fan et al., 2024). In terms of human health protection, the combination of *Lactobacillus plantarum* DP189 with galactooligosaccharides has been shown to alleviate 68% of cognitive impairments induced by MPs (Wang J. et al., 2024), presenting a novel paradigm for probiotic intervention. In industrial applications, a wet oxidation reactor has achieved a 98.6% degradation rate of MPs and biomass recovery through photothermal synergy technology (Hu et al., 2024).

## 6 Conclusion

Over the past decade, a significant volume of research has highlighted the detrimental effects of MPs and NPs exposure on human health. The evidence, pieced together from various levels of biological complexity, paints a concerning yet coherent picture. At the molecular and cellular level, *in vitro* studies have been instrumental in identifying fundamental mechanisms of toxicity, demonstrating that MPs/NPs can induce oxidative stress, mitochondrial dysfunction, and inflammatory responses in neural cells, and compromise the integrity of the blood-brain barrier. These findings are powerfully convergent with *in vivo* observations in animal models, which confirm that these cellular insults translate into functional consequences: neuroinflammation, synaptic dysfunction, and ultimately, behavioral deficits such as anxiety, cognitive impairment, and social withdrawal.

The interplay between different biological scales is particularly evident in the study of the gut-brain axis and transgenerational effects. *In vitro* models reveal how MPs disrupt gut epithelial barriers and alter microbial metabolism, while *in vivo* studies demonstrate that these gut-level disruptions propagate to the brain, affecting neurochemistry and behavior. Similarly, animal models provide direct evidence that maternal exposure leads to particle accumulation in fetal brains and results in neurodevelopmental abnormalities in offspring, a finding that gains alarming plausibility from clinical reports detecting MPs in human placental and amniotic tissues.

However, a critical gap remains between these experimental findings and direct clinical proof in humans. While epidemiological and clinical studies are beginning to corroborate the experimental data—by showing associations between MP exposure biomarkers and cognitive decline, or higher MP loads in the brains of dementia patients—they currently establish correlation, not causation. The convergence of evidence from *in vitro* mechanisms to *in vivo* phenotypes strongly suggests a genuine risk to human neurological health, but the discrepancies, particularly regarding dose-response relationships and the extrapolation of effects from high-dose, short-term animal studies to low-dose, chronic human exposure, necessitate caution. The current body of evidence, while not yet definitive for human risk assessment, unequivocally underscores the necessity of evaluating microplastic exposure as a potential environmental risk factor for neurological diseases.

In conclusion, the escalating prevalence of MPs in the environment poses a significant and plausible threat to neurological health, with potential ramifications for neurobehavioral disorders. This review highlights the urgent need for further research that strategically bridges these levels of evidence, particularly through longitudinal human cohort studies integrated with mechanistic toxicology. Simultaneously, advocating for more robust regulatory measures to diminish human exposure is a prudent and necessary step. By integrating multidisciplinary efforts across neuroscience, environmental science, and public health, we can enhance our understanding and mitigation of the risks associated with MPs, thereby safeguarding the long-term wellbeing of both human populations and ecosystems.

## 7 Review limitations

This narrative review synthesizes evidence on the impact of micro- and nanoplastics on neurobehavioral disorders by utilizing relevant search terms in scientific databases. While the article identifies several potential mechanisms, such as oxidative stress, endocrine disruption, and synaptic dysfunction, it must be acknowledged that the interconnections between these pathways are not yet fully elucidated and represent a significant challenge for the field. Furthermore, there is a notable deficiency in epidemiological studies and a lack of dose-response relationships in human cohorts, and the need for standardized methodologies in microplastic detection and exposure quantification. Additionally, the majority of the studies referenced have predominantly utilized animal models, highlighting the necessity for more direct research involving human subjects to establish causal relationships. Future research should prioritize longitudinal cohort studies with precise exposure metrics, integrated with mechanistic experiments to bridge the critical gap between animal models and human pathophysiology.
